# Cause-specific mortality in Africa and Asia: evidence from INDEPTH health and demographic surveillance system sites

**DOI:** 10.3402/gha.v7.25362

**Published:** 2014-10-29

**Authors:** P. Kim Streatfield, Wasif A. Khan, Abbas Bhuiya, Nurul Alam, Ali Sié, Abdramane B. Soura, Bassirou Bonfoh, Eliezer K. Ngoran, Berhe Weldearegawi, Momodou Jasseh, Abraham Oduro, Margaret Gyapong, Shashi Kant, Sanjay Juvekar, Siswanto Wilopo, Thomas N. Williams, Frank O. Odhiambo, Donatien Beguy, Alex Ezeh, Catherine Kyobutungi, Amelia Crampin, Valérie Delaunay, Stephen M. Tollman, Kobus Herbst, Nguyen T.K. Chuc, Osman A. Sankoh, Marcel Tanner, Peter Byass

**Affiliations:** 1 Matlab HDSS, Bangladesh; 2 International Centre for Diarrhoeal Disease Research, Bangladesh; 3 INDEPTH Network, Accra, Ghana; 4 Bandarban HDSS, Bangladesh; 5 Chakaria HDSS, Bangladesh; 6 Centre for Equity and Health Systems, International Centre for Diarrhoeal Disease Research, Bangladesh; 7 AMK HDSS, Bangladesh; 8 Centre for Population, Urbanisation and Climate Change, International Centre for Diarrhoeal Disease Research, Bangladesh; 9 Nouna HDSS, Burkina Faso; 10 Nouna Health Research Centre, Nouna, Burkina Faso; 11 Ouagadougou HDSS, Burkina Faso; 12 Institut Supérieur des Sciences de la Population, Université de Ouagadougou, Burkina Faso; 13 Taabo HDSS, Côte d'Ivoire; 14 Centre Suisse de Recherches Scientifiques en Côte d'Ivoire, Abidjan, Côte d'Ivoire; 15 Université Félix Houphoët-Boigny, Abidjan, Côte d'Ivoire; 16 Kilite-Awlaelo HDSS, Ethiopia; 17 Department of Public Health, College of Health Sciences, Mekelle University, Mekelle, Ethiopia; 18 Farafenni HDSS, The Gambia; 19 Medical Research Council, The Gambia Unit, Fajara, The Gambia; 20 Navrongo HDSS, Ghana; 21 Navrongo Health Research Centre, Navrongo, Ghana; 22 Dodowa HDSS, Ghana; 23 Dodowa Health Research Centre, Dodowa, Ghana; 24 Ballabgarh HDSS, India; 25 All India Institute of Medical Sciences, New Delhi, India; 26 Vadu HDSS, India; 27 Vadu Rural Health Program, KEM Hospital Research Centre, Pune, India; 28 Purworejo HDSS, Indonesia; 29 Department of Public Health, Universitas Gadjah Mada, Yogyakarta, Indonesia; 30 Kilifi HDSS, Kenya; 31 KEMRI-Wellcome Trust Research Programme, Kilifi, Kenya; 32 Department of Medicine, Imperial College, St. Mary's Hospital, London; 33 Kisumu HDSS, Kenya; 34 KEMRI/CDC Research and Public Health Collaboration and KEMRI Center for Global Health Research, Kisumu, Kenya; 35 Nairobi HDSS, Kenya; 36 African Population and Health Research Center, Nairobi, Kenya; 37 Karonga HDSS, Malawi; 38 Karonga Prevention Study, Chilumba, Malawi; 39 London School of Hygiene and Tropical Medicine, London, United Kingdom; 40 Niakhar HDSS, Senegal; 41 Institut de Recherche pour le Developpement (IRD), Dakar, Sénégal; 42 Agincourt HDSS, South Africa; 43 MRC/Wits Rural Public Health and Health Transitions Research Unit (Agincourt), School of Public Health, Faculty of Health Sciences, University of the Witwatersrand, Johannesburg, South Africa; 44 Umeå Centre for Global Health Research, Umeå University, Umeå, Sweden; 45 Africa Centre HDSS, South Africa; 46 Africa Centre for Health and Population Studies, University of KwaZulu-Natal, Somkhele, KwaZulu-Natal, South Africa; 47 FilaBavi HDSS, Vietnam; 48 Health System Research, Hanoi Medical University, Hanoi, Vietnam; 49 School of Public Health, Faculty of Health Sciences, University of the Witwatersrand, Johannesburg, South Africa; 50 Hanoi Medical University, Hanoi, Vietnam; 51 Swiss Tropical and Public Health Institute, Basel, Switzerland; 52 WHO Collaborating Centre for Verbal Autopsy, Umeå Centre for Global Health Research, Umeå University, Umeå, Sweden

**Keywords:** mortality, cause of death, Africa, Asia, verbal autopsy, INDEPTH Network

## Abstract

**Background:**

Because most deaths in Africa and Asia are not well documented, estimates of mortality are often made using scanty data. The INDEPTH Network works to alleviate this problem by collating detailed individual data from defined Health and Demographic Surveillance sites. By registering all deaths over time and carrying out verbal autopsies to determine cause of death across many such sites, using standardised methods, the Network seeks to generate population-based mortality statistics that are not otherwise available.

**Objective:**

To build a large standardised mortality database from African and Asian sites, detailing the relevant methods, and use it to describe cause-specific mortality patterns.

**Design:**

Individual demographic and verbal autopsy (VA) data from 22 INDEPTH sites were collated into a standardised database. The INDEPTH 2013 population was used for standardisation. The WHO 2012 VA standard and the InterVA-4 model were used for assigning cause of death.

**Results:**

A total of 111,910 deaths occurring over 12,204,043 person-years (accumulated between 1992 and 2012) were registered across the 22 sites, and for 98,429 of these deaths (88.0%) verbal autopsies were successfully completed. There was considerable variation in all-cause mortality between sites, with most of the differences being accounted for by variations in infectious causes as a proportion of all deaths.

**Conclusions:**

This dataset documents individual deaths across Africa and Asia in a standardised way, and on an unprecedented scale. While INDEPTH sites are not constructed to constitute a representative sample, and VA may not be the ideal method of determining cause of death, nevertheless these findings represent detailed mortality patterns for parts of the world that are severely under-served in terms of measuring mortality. Further papers explore details of mortality patterns among children and specifically for NCDs, external causes, pregnancy-related mortality, malaria, and HIV/AIDS. Comparisons will also be made where possible with other findings on mortality in the same regions. Findings presented here and in accompanying papers support the need for continued work towards much wider implementation of universal civil registration of deaths by cause on a worldwide basis.

The vast majority of deaths in sub-Saharan Africa and southern Asia are not individually registered nor assigned a cause of death. Data from WHO (1) show that, apart from in a few countries, the coverage of routine vital registration including cause of death in Africa and Asia is minimal and thus such cause-specific mortality data that do exist generally come from health facility records and *ad hoc* surveys. Therefore, when global estimates of cause-specific mortality are made, the data contributed from Africa and Asia are inevitably patchy and outcomes depend heavily on modelling assumptions that create huge uncertainty (2). As a result, very little is accurately known about mortality patterns in these regions, but nonetheless policy, practice, and investment decisions are made that supposedly depend on knowledge of death rates and causes.

The INDEPTH Network (International Network for the Demographic Evaluation of Populations and their Health) is an umbrella organisation for a number of independent centres operating health and demographic surveillance system (HDSS) sites, most of which are located in sub-Saharan Africa and Asia (3). These HDSS operations were started at various times and cover a range of defined rural and urban populations. Basic requirements in all the sites include registering all deaths occurring within the defined populations, and carrying out verbal autopsy (VA) procedures (interviews with relatives, care-givers, and witnesses after deaths have occurred, the results of which are subsequently interpreted into likely causes of death).

Any process of attributing cause of death, ranging from pathologists’ post-mortems through hospital cause of death records, physician certificates, and verbal autopsies, involves a combination of expertise and evidence (4). Consequently, all causes of death data also incorporate some degree of uncertainty, which may include both systematic and random variations. Undertaking VA interviews and attributing causes of death are complex processes which need to be standardised as far as is possible. A WHO-led process resulted in new standard procedures for VA in 2012 (5), in terms of defining questions that need to be included in VA interviews and VA cause of death categories corresponding to the International Classification of Diseases 10th Edition (ICD-10) (6). A detailed review of that process, building on previous VA materials, is available (7). A new version of the InterVA model for interpreting cause of death from VA data was also released in 2012 (8), which exactly corresponds to the WHO 2012 VA standard in terms of VA questions and cause of death categories.

Consideration of the absolute validity of any cause of death data is also complex. A number of studies have made comparisons between pathologists’ post-mortems and hospital records (9–11); others have compared validity between hospital records and mortality registers (12), with varying degrees of concordance. In some cases, VA findings have been compared with hospital records, but this approach has been hampered by the generally small and unrepresentative proportions of deaths actually occurring in hospitals located in populations where VAs are used ( 13–15). Many studies have compared the use of automated VA coding models with results from physicians coding the same VA material (often termed physician-coded verbal autopsy (PCVA)), several of which have involved using InterVA models (16). However, there can be difficulties in separating differences that arise from possible systematic errors in models and more random inter- or intra-physician variations (17). For some specific causes of death, there can be absolute standards for comparison, for example, *ante-mortem* HIV or sickle cell status, but this only applies to a minority of causes (18, 19). Many cause of death processes do not allow the attribution of uncertainty at the individual level. Although assigning a death as being 100% due to a particular cause simplifies further analyses, in reality there is a range of certainty associated with individual cause of death assignments, depending on the extent of available information for a particular case, as well as other factors. The probabilistic modelling used by InterVA-4 facilitates the capture of this uncertainty for each individual case, with the possibility of attributing part of a death as being of indeterminate cause (8).

Despite the absence of widespread and reliable cause of death registration across Africa and Asia, much can be learnt about mortality patterns by considering standardised VA findings from sites where such data are routinely collected at the population level on a longitudinal basis. Mortality surveillance within circumscribed populations leads to findings based on every death that occurs. Consequently, cause-specific fractions total 100%, without the difficulties that some modelled estimates have encountered of needing to impose an overall mortality envelope. Furthermore, the advantages of consistency over time and place offered by VA models in assigning cause of death is particularly relevant for large epidemiological studies such as reported here, even though physicians might arguably follow a more nuanced approach in assigning individual causes of death.

The objectives of this introductory paper are to describe a large VA dataset compiled across a range of INDEPTH HDSS sites in Africa and Asia together with details of the overall methods used, as well as to report key findings on overall patterns of mortality and highlight areas of specific interest which have been examined in more detail in accompanying papers. Specifically, childhood mortality (20) and adult non-communicable disease (NCD) mortality (21), plus mortality from external causes (22) and associated with pregnancy (23), have been explored in more detail. Malaria (24) and HIV/AIDS-related (25) mortality, being two highly significant causes that vary considerably between sites, are also documented separately. Overall, findings and ways forward are brought together in a concluding synthesis (26).

The publication of this series of papers coincides with depositing the overall cause of death dataset (27) into the public domain at the INDEPTH Data Repository (28). Half of the HDSSs involved are already part of INDEPTH's iSHARE programme (www.indepth-ishare.org), and already have other individual-level population surveillance data in the public domain. The remaining sites have aggregated population data publicly available as part of the associated INDEPTHStats programme. As agreed by the INDEPTH Network Board, a separate set of anonymised identifiers have been used for the public domain cause of death data as distinct from other individual-level data, in order to guard against identity disclosure risks. Enquiries relating to specific research plans that would need to link the individual cause of death data with other individual-level data can be made to the INDEPTH Secretariat.

## Populations and methods

**Fig. 1:**
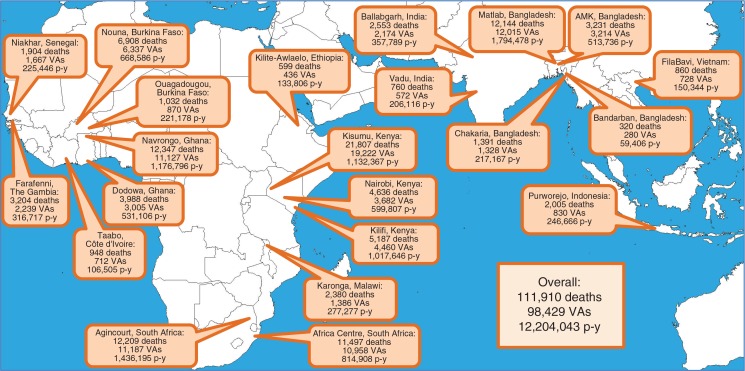
Map showing locations of the 22 sites, with numbers of deaths registered, VAs completed, and person-years observed.

Cause of death data based on VA interviews were contributed by 14 INDEPTH HDSS sites in sub-Saharan Africa and eight sites in Asia, located as shown in Fig. 1. The principles of the Network and its constituent population surveillance sites have been described generically (3), and detailed descriptions of the individual sites involved, including local attributes, are available elsewhere (29–50). Each HDSS site is committed to long-term longitudinal surveillance of circumscribed populations, typically each covering around 50,000–100,000 people. Households are registered and visited regularly by lay field-workers, with a frequency varying from once per year to several times per year. All vital events are registered at each such visit, and any deaths recorded are followed up with VA interviews, usually undertaken by specially trained lay interviewers. A few sites were already operational in the 1990s, but in this dataset 95% of the person-time observed related to the period from 2000 onwards, with 68% from 2006 onwards. Two sites, in Nairobi and Ouagadougou, followed urban populations, while the remainder covered areas that were generally more rural in character, although some included local urban centres. Sites covered entire populations, although the Karonga, Malawi, site only contributed VAs for deaths of people aged 12 years and older. Because the sites were not located or designed in a systematic way to be representative of national or regional populations, it is not meaningful to aggregate results over sites. Therefore, site-specific, cause-specific mortality fractions (CSMFs) and mortality rates (CSMRs) were used as the basis for analyses and comparisons. Since each site encompassed an entire non-sampled population, it was not meaningful to consider confidence intervals around site-specific measurements. Uncertainty around individual assignments of cause of death was however incorporated into the dataset as described below.

Because there are possible inter-site and inter-year variations in the age–sex structure of the populations concerned, it was also important, at least for some comparisons, to be able to adjust CSMFs and CSMRs to a standard population. Due to the different age and sex profiles of many causes of death, if this was not done then observed variations in CSMFs and CSMRs could have been due to (or masked by) differences in population structure between sites and over time. For this purpose, we took the INDEPTH 2013 standard population structure for low- and middle-income countries (LMICs) in Africa and Asia (51), as shown in Table 1. This public-domain standard population has been presented in relation to other global standards such as Segi and WHO, from which it differs in reflecting the higher fertility and younger-age mortality rates commonly seen in LMIC populations (51). As shown in Table 1, this standard has a very similar structure to the aggregated population from which these VA data came. Using the INDEPTH 2013 standard, encompassing lower income populations in both Africa and Asia, meant that the overall effect of standardisation across the whole mortality dataset was minimal, while resulting in important adjustments for certain sub-groups. Thus, calculating a standardised weight for every combination of site, age group, sex, and year made it possible to compare cause-specific mortality without concern for differences in underlying population structures (referred to hereafter as age–sex–time standardisation). Using the same INDEPTH 2013 standard, it will be possible to directly compare future work on these same lines with any mortality data from Africa and Asia.

**Table 1:** Adapted INDEPTH 2013 standard population (51) by age group and sex, as percentage of total population (used for calculating adjusted cause-specific fractions and rates), compared with observed population structures in this dataset, overall and for African sites and Asian sites

	INDEPTH 2013 standard		All sites in this dataset		African sites in this dataset		Asian sites in this dataset	
	
Age group	Male (%)	Female (%)	Male (%)	Female (%)	Male (%)	Female (%)	Male (%)	Female (%)
0–28 days	0.11	0.10	0.11	0.11	0.12	0.12	0.08	0.08
1–11 months	1.49	1.38	1.33	1.31	1.46	1.45	1.03	0.98
1–4 years	6.01	5.57	5.62	5.48	6.07	5.99	4.51	4.24
5–14 years	13.76	12.57	12.78	12.44	13.50	13.24	11.01	10.48
15–49 years	22.54	23.50	22.62	25.39	21.74	24.71	24.79	27.05
50–64 years	3.87	4.36	3.61	4.40	3.11	3.11	4.84	5.16
65+ years	2.22	2.52	2.18	2.62	1.96	1.96	2.73	3.04

All causes of death assignments in this dataset were made using the InterVA-4 model version 4.02 (8). InterVA-4 uses probabilistic modelling to arrive at likely cause(s) of death for each VA case, the workings of the model being based on a combination of expert medical opinion and relevant available data. InterVA-4 is the only model currently available that processes VA data according to the WHO 2012 standard and categorises causes of death according to ICD-10. Since the VA data reported here were collected before the WHO 2012 standard was formulated, they were all retrospectively transformed into the WHO 2012 and InterVA-4 input format for processing. The phrase ‘successfully completed VA interview’ means that a VA interview was undertaken which yielded basic personal characteristics of the deceased (age, sex, etc.) and some symptoms relating to the final illness. The InterVA-4 ‘high’ malaria setting was used for all the West African sites, plus the East African sites (with the exceptions, on the grounds of high altitude, of Nairobi, Kenya, and Kilite-Awlaelo, Ethiopia); other sites used the ‘low’ setting. The InterVA-4 ‘high’ HIV/AIDS setting was used for sites in Kenya, Malawi, and South Africa; for all other sites the ‘low’ setting was used. These settings were chosen in line with InterVA recommendations and previous experience, and are discussed further in the specific papers on malaria and HIV/AIDS-related mortality (24, 25).

The InterVA-4 model was applied to the data from each site, yielding, for each case, up to three possible causes of death or an indeterminate result. In a minority of cases, for example, where symptoms were vague, contradictory or mutually inconsistent, it was impossible for InterVA-4 to determine a cause of death, and these deaths were attributed as entirely indeterminate. For the remaining cases, one to three likely causes and their likelihoods were assigned by InterVA-4, and if the sum of their likelihoods was less than one, the residual component was then assigned as being indeterminate. This was an important process for capturing uncertainty in cause of death outcome(s) from the model at the individual level, thus avoiding over-interpretation of specific causes. As a consequence there were three sources of unattributed cause of death: deaths registered for which VAs were not successfully completed; VAs completed but where the cause was entirely indeterminate; and residual components of deaths attributed as indeterminate.

An overall dataset (27) was compiled in which each case had between one and four records, each with its own cause and likelihood. Cases for which VAs were not successfully completed had single records with the cause of death recorded as ‘VA not completed’ and a likelihood of one. Thus, the overall sum of the likelihoods equated to the total number of deaths. Each record also contained a population weighting factor reflecting the ratio of the population fraction for its site, age group, sex, and year to the corresponding age group and sex fraction in the standard population described in Table 1, for the purposes of standardisation. Then a further factor was calculated for each record as the product of the VA cause likelihood and the population standard weighting (both described above), which could be used as the basis for calculating age–sex–time standardised CSMFs and CSMRs.

These descriptions of methods used to construct this multisite dataset (27) apply to the following series of analytical papers using the dataset (20–25). A standard Box summarising these methods is included in each of these papers.

In this context, all of these data are secondary datasets derived from primary data collected separately by each participating site. In all cases, the primary data collection was covered by site-level ethical approvals relating to on-going health and demographic surveillance in those specific locations. No individual identity or household location data were included in the secondary data and no specific ethical approvals were required for these pooled analyses.

## Results

A total of 111,910 deaths occurring over 12,204,043 person-years were registered across the 22 sites. For 98,429 of these deaths (88.0%), VAs were successfully completed. Figure 1 includes the numbers of deaths, completed VAs and person-time observed for each site. Among the 98,429 completed VAs, InterVA-4 was unable to reach any conclusive cause of death (i.e. arrived at 100% indeterminate outcome) in 4,680 (4.8%) of cases. Residual indeterminate fractions totalled 7,545.9 (7.7%) of completed VAs. Thus, out of the total of 111,910 deaths recorded, specific causes were successfully assigned to 86,203 deaths (77.0%). Age–sex–time standardisation made less than 1% difference to the overall dataset (112,653 standardised deaths compared with 111,910 observed deaths) but was particularly important for some sites, for example, the urban slum population in Nairobi, where the population structure differed markedly from the standard.

**Fig. 2:**
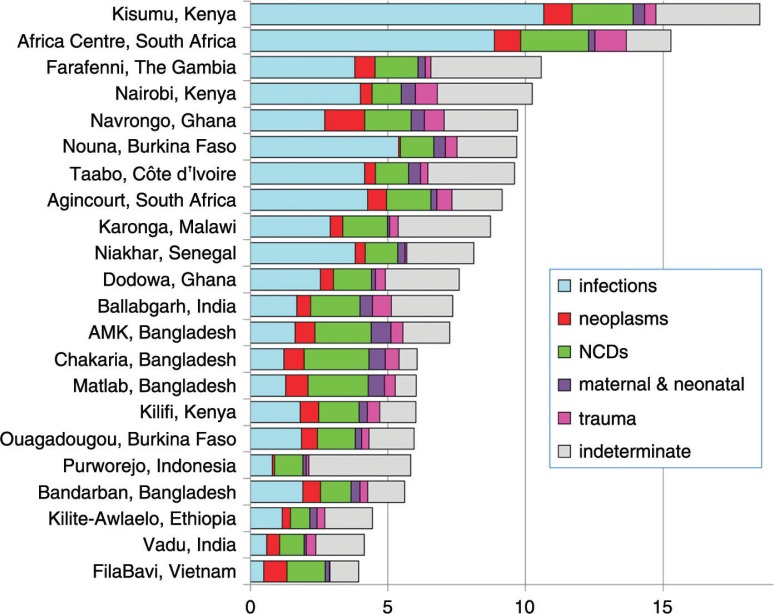
Age–sex–time-standardised mortality rates per 1,000 person-years by cause group and site for a total of 111,910 deaths over 12,204,043 person-years observed.

Table 2 shows mortality rates per 1,000 person-years by age group and time period for each of the 22 participating sites. Figure 2 shows age–sex–time standardised mortality rates for each site, by major categories of cause of death (infections, neoplasms, NCDs, maternal/neonatal, trauma, and indeterminate). The indeterminate category includes cases where VAs were not done, as well as indeterminate components reflecting individual uncertainty in cause of death assignment. All-cause age–sex–time standardised mortality rates in individual sites ranged from 18.5 per 1,000 person-years in Kisumu, Kenya, to 3.9 per 1,000 person-years in FilaBavi, Vietnam. A large part of this variation was accounted for by differences in infectious causes of death (10.7 per 1,000 person-years in Kisumu to 0.5 per 1,000 person-years in FilaBavi). A number of sites reflected low overall mortality rates as a consequence of being at stages of demographic transition where life expectancy increases as health standards improve, but where population proportions of elderly people remain relatively low.

**Table 2:** Mortality rates per 1,000 person-years by site, period, and age group for a total of 111,910 deaths observed over 12,204,043 person-years at INDEPTH sites in Africa and Asia

		0–28 days		1–11 months		1–4 years		5–14 years		15–49 years		50–64 years		65+ years	
		
Site	Period	Male	Female	Male	Female	Male	Female	Male	Female	Male	Female	Male	Female	Male	Female
Matlab, Bangladesh	2000–05	471.1	417.3	10.6	13.3	3.5	3.2	0.8	0.9	2.2	1.5	14.1	8.0	65.9	57.3
	2006–12	379.8	272.1	7.5	8.5	2.6	2.7	0.6	0.5	1.9	1.3	14.0	7.2	62.7	54.9
Bandarban, Bangladesh	2006–12	265.5	70.6	38.9	17.8	0.7	3.1	1.5	0.6	3.0	2.5	10.0	9.7	43.1	35.7
Chakaria, Bangladesh	2006–12	506.4	405.5	11.6	22.0	3.9	4.0	1.1	0.9	2.0	1.8	12.4	13.2	64.0	68.9
AMK, Bangladesh	2000–05	454.2	325.4	9.4	14.2	3.4	3.1	0.8	0.8	1.8	1.6	11.5	7.0	64.4	59.1
	2006–12	413.9	298.1	8.1	13.2	2.3	2.2	0.7	0.5	2.1	1.4	11.8	8.3	68.3	55.5
Nouna, Burkina Faso	1992–99	114.1	87.6	39.8	38.8	28.1	31.6	8.1	4.3	8.1	5.4	21.7	28.7	92.5	56.7
	2000–05	148.0	136.4	43.0	41.9	20.0	18.4	2.8	2.5	4.7	4.4	18.2	13.2	65.1	64.6
	2006–12	101.4	84.3	24.6	24.2	14.4	11.1	1.7	1.5	3.3	2.2	15.8	10.6	60.5	43.3
Ouagadougou, Burkina Faso	2006–12	167.2	106.9	20.5	21.2	8.1	7.4	1.5	1.2	2.0	1.9	15.9	7.5	51.9	32.5
Taabo, Côte d'Ivoire	2006–12	202.7	198.8	29.6	34.3	15.4	15.0	1.7	1.8	5.4	4.8	16.4	11.7	61.4	45.6
Kilite-Awaleo, Ethiopia	2006–12	244.2	130.0	12.8	12.4	3.4	2.3	1.2	0.9	2.2	1.8	6.2	6.5	36.4	32.6
Farafenni, The Gambia	1992–99	333.3	320.0	47.5	76.2	42.2	22.4	7.3	4.0	5.9	5.2	34.7	18.6	97.0	67.2
	2000–05	202.2	212.9	35.1	29.0	12.9	13.1	2.1	2.1	4.9	4.4	24.8	14.9	80.3	64.9
	2006–12	286.8	127.8	22.5	17.8	6.4	6.0	1.8	1.2	4.5	3.7	21.7	11.7	85.7	53.5
Navrongo, Ghana	2000–05	327.5	283.3	46.1	40.8	11.6	11.3	2.6	1.7	6.6	4.8	33.7	18.1	74.7	61.3
	2006–12	240.2	179.4	22.8	21.2	8.5	7.9	2.0	1.5	6.0	3.2	28.6	13.7	69.6	49.7
Dodowa, Ghana	2006–12	115.7	63.9	7.9	9.6	4.9	4.6	1.4	1.3	4.8	4.6	20.6	14.5	62.6	50.4
Ballabgarh, India	2006–12	274.8	286.1	21.8	27.6	3.2	4.9	0.7	1.0	3.4	1.9	19.0	9.2	75.3	54.0
Vadu, India	2006–12	88.6	117.4	3.7	4.4	1.5	1.2	0.5	0.0	1.8	1.2	12.2	8.1	43.3	43.4
Purworejo, Indonesia	2000–05	212.3	132.2	10.9	6.9	2.4	2.4	0.9	0.5	2.1	1.6	15.5	13.6	60.9	54.0
Kilifi, Kenya	2006–12	176.4	143.4	10.0	9.1	2.8	2.3	0.9	0.7	3.0	2.9	16.4	10.4	61.2	42.5
Kisumu, Kenya	2000–05	298.4	307.0	110.7	112.7	32.3	31.1	2.5	2.8	18.0	19.0	40.6	22.7	75.4	52.7
	2006–12	260.9	224.9	75.6	72.9	22.6	23.0	2.6	2.2	12.0	11.4	30.3	18.7	75.5	56.3
Nairobi, Kenya	2000–05	428.4	320.9	67.3	48.8	10.0	6.9	2.4	1.8	9.6	5.6	39.9	10.6	55.2	36.0
	2006–12	342.5	299.3	51.0	48.6	7.1	5.8	1.1	1.0	8.2	4.0	35.9	6.6	48.8	40.4
Karonga, Malawi	2000–05	354.5	228.4	34.4	27.0	9.1	9.2	2.3	1.0	8.3	9.4	23.3	15.7	48.9	54.3
	2006–12	293.5	219.8	23.2	21.9	8.0	6.9	1.2	1.4	4.9	4.2	18.4	11.5	45.5	44.5
Niakhar, Senegal	2000–05	235.9	184.7	37.1	25.6	18.4	22.6	3.4	3.0	5.1	5.8	13.6	15.3	99.6	79.8
	2006–12	148.1	105.1	17.2	16.3	9.6	10.2	1.9	1.1	3.3	2.8	13.9	8.2	67.1	49.2
Agincourt, South Africa	1992–99	103.6	58.0	12.0	15.1	4.4	4.4	0.8	0.7	3.8	3.0	13.0	10.6	30.6	56.7
	2000–05	133.3	105.9	32.5	27.9	7.7	6.3	1.0	0.9	8.7	8.9	23.7	20.9	34.4	70.7
	2006–12	159.1	150.2	31.7	30.1	5.7	4.9	1.5	1.2	9.6	8.6	24.4	25.1	33.2	84.0
Africa Centre, South Africa	2000–05	169.7	132.0	49.5	49.5	9.0	8.8	1.6	1.8	18.7	16.9	46.4	22.6	88.2	49.6
	2006–12	56.1	49.9	28.7	26.3	5.3	4.1	1.3	1.1	12.9	11.1	46.2	21.2	82.3	52.4
FilaBavi, Vietnam	2006–12	144.5	92.3	1.2	4.3	1.1	0.8	0.6	0.1	3.0	1.2	9.6	3.1	50.7	33.3

## Discussion

This dataset documents individual deaths across sub-Saharan Africa and southern Asia on a hitherto unprecedented scale. In addition, because the deaths are recorded in the context of longitudinal surveillance operations, it is also possible to determine population-based mortality rates, overall and by year, cause, age group, and sex. The application of the WHO 2012 VA standard (7) and the InterVA-4 (8) model to these data enabled assignment of cause of death in a consistent manner, and, where appropriate, age–sex–time standardisation of rates enabled systematic comparisons between sites.

This dataset provided opportunities for a wide range of more detailed analyses, as reflected in the following series of papers. Apart from looking in more detail at causes of death within obvious population sub-groups such as neonates, infants, and under-5s (20), it is also interesting to consider the detail within some of the cause groups shown in Figure 2. It would appear that population-based rates of NCD mortality are relatively constant across the sites, compared with the variations in overall mortality, which seem to be more strongly driven by the magnitude of infectious causes (21). Although there is much reported on so-called epidemics of NCDs in LMICs, this may partly reflect relatively large proportions of NCD mortality in populations also experiencing historically low levels of overall mortality, particularly in Asia. These low overall rates are demographically driven in populations experiencing rapid life expectancy increases, but not yet having accumulated larger proportions of older people. Nevertheless, concerns over current accumulations of NCD risk factors are very valid in relation to future NCD mortality. These results on NCD mortality may therefore constitute an important baseline measurement against which to judge future developments. External causes of death – intentional and unintentional, self-inflicted and imposed – also constitute an increasingly large component of mortality in various places and age groups, which have been explored further in this dataset (22). This was also a sufficiently large dataset to look in more detail at some specific causes of death, such as pregnancy-related deaths (23) and infectious causes such as malaria (24) and HIV/AIDS (25). Availability of the dataset in the public domain will facilitate further analyses of specific causes and patterns of mortality.

Although HDSS sites such as those contributing data here can be critiqued in terms of how representative they may be of surrounding areas, this consideration has to be offset against the unique opportunities HDSSs have of assessing mortality patterns for complete communities, rather than the more commonly used health facility mortality records. Most deaths in Africa and Asia do not occur in health facilities, and it is by no means evident that facility-based deaths are very representative of mortality in general. Cause of death as determined by VA may also be regarded as less than ideal, but it is the only viable method for large-scale cause of death assignment in Africa and Asia. Here, the WHO 2012 VA standard and the InterVA-4 model have been used to ensure, as far as possible, that there is consistency across the sites and time periods involved, which is not guaranteed with physician interpretation of VA. Many sites also undertake physician interpretation of their VAs, which may differ in detail from these results, and be reported separately; we are not making any comparisons with physician findings here. We acknowledge that it was a compromise to have to transform VA data collected using a range of historical instruments, but that was unavoidable. Most of the instruments used had evolved from earlier WHO and INDEPTH VA versions with much common core content, which were themselves the starting point for the development of the WHO 2012 standard. The application of age–sex–time standardisation, using the INDEPTH 2013 population standard, further enabled comparisons of mortality over time and place to be made objectively.

A Population Health Metrics Research Consortium (PHMRC) study aimed to collect a ‘gold standard’ VA dataset from selected tertiary institutions, which has been used both to build VA models and evaluate different approaches to VA cause of death assignment (52). Although that study concluded that InterVA-4 was less effective than PHMRC methods in assigning cause of death, that conclusion was reached by comparing the internal validity of PHMRC methods within the ‘gold standard’ dataset against the external validity of InterVA-4 and physician coding (53). Issues with the quality of the PHMRC VA data, the use of different VA questions and deviations from ICD-10 classifications further compromised those findings, but nevertheless InterVA-4 coding of the PHMRC data still demonstrated an overall concordance correlation of 0.61. Since InterVA-4 is the only available VA model which corresponds to the WHO 2012 VA standard and ICD-10 coded causes of death, it was the preferred choice to use here.

A number of sites contributing data to the overall dataset have undertaken site-specific analyses of their mortality patterns which are reported separately (54–66). In some countries (Bangladesh, Ghana, Burkina Faso, Kenya, South Africa) there were multiple sites involved which present interesting opportunities to consider within-country variations. This also facilitates comparisons with other national sources of data such as Demographic and Health Surveys and Global Burden of Disease outputs. To some extent, it was also possible to look at trends in mortality over time, although that was limited by the different time periods over which individual sites have been operating. Since more sites have reported for recent periods, there may be findings here of interest in terms of trends towards the 2015 deadlines for Millennium Development Goals. These may also serve as baseline figures for post-2015 targets.

Most of the detailed comparisons made between results from this dataset and comparable figures from various other sources of estimates, explored in the accompanying papers, showed a high degree of congruence. Given that the methodologies involved – of counting individual deaths at INDEPTH sites and aggregating upwards, contrasted with taking available data sources and constructing global models to derive national estimates – are completely different, this congruence in findings adds plausibility to both approaches. Nevertheless, it must still be recognised that moving towards complete civil registration of deaths, including cause of death, is a critical objective yet to be achieved in Africa and Asia (67).

## References

[1] World Health Organization Civil registration coverage of cause of death (%), 2005–2011. http://www.who.int/gho/mortality_burden_disease/registered_deaths/mbd_018.jpg.

[2] Byass P, de Courten M, Graham WJ, Laflamme L, McCaw-Binns A, Sankoh OA (2013). Reflections on the global burden of disease 2010 estimates. PLoS Med.

[3] Sankoh O, Byass P (2012). The INDEPTH Network: filling vital gaps in global epidemiology. Int J Epidemiol.

[4] Byass P (2011). Whither verbal autopsy?. Popul Health Metr.

[5] World Health Organization (2012). Verbal autopsy standards: the 2012 WHO verbal autopsy instrument.

[6] World Health Organization (2011). International statistical classification of diseases and related health problems. Instruction Manual.

[7] Leitao J, Chandramohan D, Byass P, Jakob R, Bundhamcharoen K, Choprapawon C (2013). Revising the WHO verbal autopsy instrument to facilitate routine cause-of-death monitoring. Glob Health Action.

[8] Byass P, Chandramohan D, Clark SJ, D'Ambruoso L, Fottrell E, Graham WJ (2012). Strengthening standardised interpretation of verbal autopsy data: the new InterVA-4 tool. Glob Health Action.

[9] Swaro A, Adhiyaman V (2010). Autopsy in older medical patients: concordance in ante- and post-mortem findings and changing trends. J R Coll Physicians Edinb.

[10] Gulsvik AK, Gulsvik A, Svendsen E, Mæhle BO, Thelle DS, Wyller TB (2011). Diagnostic validity of fatal cerebral strokes and coronary deaths in mortality statistics: an autopsy study. Eur J Epidemiol.

[11] Ordi J, Ismail MR, Carrilho C, Romagosa C, Osman N, Machungo F (2009). Clinico-pathological discrepancies in the diagnosis of causes of maternal death in sub-Saharan Africa: retrospective analysis. PLoS Med.

[12] Herrett E, Shah AD, Boggon R, Denaxas S, Smeeth L, van Staa T (2013). Completeness and diagnostic validity of recording acute myocardial infarction events in primary care, hospital care, disease registry and national mortality records: cohort study. Br Med J.

[13] Kahn K, Tollman SM, Garenne M, Gear JS (2000). Validation and application of verbal autopsies in a rural area of South Africa. Trop Med Int Health.

[14] Misganaw A, Mariam DH, Araya T, Aneneh A (2012). Validity of verbal autopsy method to determine causes of death among adults in the urban setting of Ethiopia. BMC Med Res Methodol.

[15] Aggarwal A, Kumar P, Pandit S, Kumar R (2013). Accuracy of WHO verbal autopsy tool in determining major causes of neonatal deaths in India. PLoS One.

[16] Leitao J, Desai N, Aleksandrowicz L, Byass P, Miasnikof P, Tollman S (2014). Comparison of physician-certified verbal autopsy with computer-coded verbal autopsy for cause of death assignment in hospitalized patients in low- and middle-income countries: systematic review. BMC Med.

[17] Byass P, Kahn K, Fottrell E, Mee P, Collinson MA, Tollman SM (2011). Using verbal autopsy to track epidemic dynamics: the case of HIV-related mortality in South Africa. Popul Health Metr.

[18] Byass P, Calvert C, Miiro-Nakiyingi J, Lutalo T, Michael D, Crampin A (2013). InterVA-4 as a public health tool for measuring HIV/AIDS mortality: a validation study from five African countries. Glob Health Action.

[19] Ndila C, Bauni E, Nyirongo V, Mochamah G, Makazi A, Kosgei P (2014). Verbal autopsy as a tool for identifying children dying of sickle cell disease: a validation study conducted in Kilifi district, Kenya. BMC Med.

[20] Streatfield PK, Khan WA, Bhuiya A, Hanifi SMA, Alam N, Ouattara M (2014). Cause-specific childhood mortality in Africa and Asia: evidence from INDEPTH Health and Demographic Surveillance System sites. Glob Health Action.

[21] Streatfield PK, Khan WA, Bhuiya A, Hanifi SMA, Alam N, Bagagnan C (2014). Adult non-communicable disease mortality in Africa and Asia: evidence from INDEPTH Health and Demographic Surveillance System sites. Glob Health Action.

[22] Streatfield PK, Khan WA, Bhuiya A, Hanifi SMA, Alam N, Diboulo E (2014). Mortality from external causes in Africa and Asia: evidence from INDEPTH Health and Demographic Surveillance System sites. Glob Health Action.

[23] Streatfield PK, Alam N, Compaore Y, Rossier C, Soura AB, Bonfoh B (2014). Pregnancy-related mortality in Africa and Asia: evidence from INDEPTH Health and Demographic Surveillance System sites. Glob Health Action.

[24] Streatfield PK, Khan WA, Bhuiya A, Hanifi SMA, Alam N, Diboulo E (2014). Malaria mortality in Africa and Asia: evidence from INDEPTH Health and Demographic Surveillance System sites. Glob Health Action.

[25] Streatfield PK, Khan WA, Bhuiya A, Hanifi SMA, Alam N, Millogo O (2014). HIV/AIDS-related mortality in Africa and Asia: evidence from INDEPTH Health and Demographic Surveillance System sites. Glob Health Action.

[26] Sankoh O, Byass P (2014). Cause-specific mortality at INDEPTH Health and Demographic Surveillance System Sites in Africa and Asia: concluding synthesis. Glob Health Action.

[27] INDEPTH Network (2014). INDEPTH Network Cause-Specific Mortality – Release 2014. http:\\www.indepth-network.org.

[28] Sankoh O, Herbst AJ, Juvekar S, Tollman S, Byass P, Tanner M (2013). INDEPTH launches a data repository and INDEPTHStats. Lancet Glob Health.

[29] Razzaque A, Nahar L, Akter Khanam M, Streatfield PK (2010). Socio-demographic differentials of adult health indicators in Matlab, Bangladesh: self-rated health, health state, quality of life and disability level. Glob Health Action.

[30] INDEPTH Network Bandarban HDSS. http://www.indepth-network.org/Profiles/Bandarban%20HDSS.pdf.

[31] Hanifi MA, Mamun AA, Paul A, Hasan SA, Hoque S, Sharmin S (2012). Profile: the Chakaria Health and Demographic Surveillance System. Int J Epidemiol.

[32] Lindeboom W, Das SC, Ashraf A (2011). Health and demographic surveillance report 2009 – Abhoynagar and Mirsarai.

[33] Sié A, Louis VR, Gbangou A, Müller O, Niamba L, Stieglbauer G (2010). The health and demographic surveillance system (HDSS) in Nouna, Burkina Faso, 1993–2007. Glob Health Action.

[34] Rossier C, Soura A, Baya B, Compaoré G, Dabiré B, Dos Santos S (2012). Profile: the Ouagadougou Health and Demographic Surveillance System. Int J Epidemiol.

[35] Kouadio MK, Righetti AA, Abé NN, Wegmüller R, Weiss MG, N'goran EK (2013). Local concepts of anemia-related illnesses and public health implications in the Taabo health demographic surveillance system, Côte d'Ivoire. BMC Hematol.

[36] Weldearegawi B, Ashebir Y, Gebeye E, Gebregziabiher T, Yohannes M, Mussa S (2013). Emerging chronic non-communicable diseases in rural communities of Northern Ethiopia: evidence using population-based verbal autopsy method in Kilite Awlaelo surveillance site. Health Policy Plan.

[37] Oduro AR, Wak G, Azongo D, Debpuur C, Wontuo P, Kondayire F (2012). Profile: the Navrongo Health and Demographic Surveillance System. Int J Epidemiol.

[38] Gyapong M, Sarpong D, Awini E, Manyeh AK, Tei D, Odonkor G (2013). Profile: the Dodowa Health and Demographic Surveillance System. Int J Epidemiol.

[39] Jasseh M, Webb EL, Jaffar S, Howie S, Townend J, Smith PG (2011). Reaching millennium development goal 4 – the Gambia. Trop Med Int Health.

[40] Ng N, Hakimi M, Santosa A, Byass P, Wilopo SA, Wall S (2012). Is self-rated health an independent index for mortality among older people in Indonesia?. PLoS One.

[41] Kant S, Misra P, Gupta S, Goswami K, Krishnan A, Nongkynrih B (2013). Profile: the Ballabgarh Health and Demographic Surveillance System (CRHSP-AIIMS). Int J Epidemiol.

[42] Hirve S, Juvekar S, Sambhudas S, Lele P, Blomstedt Y, Wall S (2012). Does self-rated health predict death in adults aged 50 years and above in India? Evidence from a rural population under health and demographic surveillance. Int J Epidemiol.

[43] Scott JA, Bauni E, Moisi JC, Ojal J, Gatakaa H, Nyundo C (2012). Profile: the Kilifi Health and Demographic Surveillance System (KHDSS). Int J Epidemiol.

[44] Odhiambo FO, Laserson KF, Sewe M, Hamel MJ, Feikin DR, Adazu K (2012). Profile: the KEMRI/CDC Health and Demographic Surveillance System – Western Kenya. Int J Epidemiol.

[45] Oti SO, Mutua M, Mgomella GS, Egondi T, Ezeh A, Kyobutungi C (2013). HIV mortality in urban slums of Nairobi, Kenya 2003–2010: a period effect analysis. BMC Public Health.

[46] Crampin AC, Dube A, Mboma S, Price A, Chihana M, Jahn A (2012). Profile: the Karonga Health and Demographic Surveillance System. Int J Epidemiol.

[47] Delaunay V, Douillot L, Diallo A, Dione D, Trape JF, Medianikov O (2013). Profile: the Niakhar Health and Demographic Surveillance System. Int J Epidemiol.

[48] Kahn K, Collinson MA, Gómez-Olivé FX, Mokoena O, Twine R, Mee P (2012). Profile: Agincourt health and socio-demographic surveillance system. Int J Epidemiol.

[49] Herbst AJ, Mafojane T, Newell ML (2011). Verbal autopsy-based cause-specific mortality trends in rural KwaZulu-Natal, South Africa, 2000–2009. Popul Health Metr.

[50] Huong DL, Minh HV, Vos T, Janlert U, Van DD, Byass P (2006). Burden of premature mortality in rural Vietnam from 1999–2003: analyses from a Demographic Surveillance site. Popul Health Metr.

[51] Sankoh O, Sharrow D, Herbst K, Kabudula CW, Alam N, Kant S (2014). The INDEPTH standard population for low- and middle-income countries, 2013. Glob Health Action.

[52] Murray CJ, Lozano R, Flaxman AD, Serina P, Phillips D, Stewart A (2014). Using verbal autopsy to measure causes of death: the comparative performance of existing methods. BMC Med.

[53] Byass P (2014). Usefulness of the Population Health Metrics Research Consortium gold standard verbal autopsy data for general verbal autopsy methods. BMC Med.

[54] Alam N, Chowdhury HR, Ahmed A, Rahman M, Streatfield PK (2014). Distribution of cause of death in rural Bangladesh during 2003–2010: evidence from two rural areas within Matlab Health and Demographic Surveillance site. Glob Health Action.

[55] Hanifi SMA, Mahmood SS, Bhuiya A (2014). Cause-specific mortality and socioeconomic status in Chakaria, Bangladesh. Glob Health Action.

[56] Alam N, Chowdhury HR, Das SC, Ashraf A, Streatfield PK (2014). Causes of death in two rural demographic surveillance sites in Bangladesh, 2004–2010: automated coding of verbal autopsies using InterVA-4. Glob Health Action.

[57] Soura AB, Lankoande B, Millogo R, Bangha M (2014). Comparing causes of death between formal and informal neighborhoods in urban Africa: evidence from Ouagadougou Health and Demographic Surveillance System. Glob Health Action.

[58] Weldearegawi B, Melaku YA, Spigt M, Dinant GJ (2014). Applying the InterVA-4 model to determine causes of death in rural Ethiopia. Glob Health Action.

[59] Awini E, Sarpong D, Adjei A, Manyeh AK, Amu A, Akweongo P (2014). Estimating cause of adult (15+ years) death using InterVA-4 in a rural district of southern Ghana. Glob Health Action.

[60] Jasseh M, Howie SRC, Gomez P, Scott S, Roca A, Cham M (2014). Disease-specific mortality burdens in a rural Gambian population using verbal autopsy, 1998–2007. Glob Health Action.

[61] Rai SK, Kant S, Misra P, Srivastava R, Pandav CS (2014). Cause of death during 2009–2012, using a probabilistic model (InterVA-4): an experience from Ballabgarh Health and Demographic Surveillance System in India. Glob Health Action.

[62] Ndila C, Bauni E, Mochamah G, Nyirongo V, Makazi A, Kosgei P (2014). Causes of death among persons of all ages within the Kilifi Health and Demographic Surveillance System, Kenya, determined from verbal autopsies interpreted using the InterVA-4 model. Glob Health Action.

[63] Amek NO, Odhiambo FO, Khagayi S, Moige H, Orwa G, Hamel MJ (2014). Childhood cause-specific mortality in rural Western Kenya: application of the InterVA-4 model. Glob Health Action.

[64] Oti SO, van de Vijver S, Kyobutungi C (2014). Trends in non-communicable disease mortality among adult residents in Nairobi's slums, 2003–2011: applying InterVA-4 to verbal autopsy data. Glob Health Action.

[65] Kabudula CW, Tollman S, Mee P, Ngobeni S, Silaule B, Gómez-Olivé FX (2014). Two decades of mortality change in rural northeast South Africa. Glob Health Action.

[66] Mossong J, Byass P, Herbst K (2014). Who died of what in rural KwaZulu-Natal, South Africa: a cause of death analysis using InterVA-4. Glob Health Action.

[67] Byass P (2012). The UN needs joined-up thinking on vital registration. Lancet.

